# Switchgrass (*Panicum virgatum* L.) Genotypes Differ between Coastal Sites and Inland Road Corridors in the Northeastern US

**DOI:** 10.1371/journal.pone.0130414

**Published:** 2015-06-30

**Authors:** Geoffrey Ecker, Juan Zalapa, Carol Auer

**Affiliations:** 1 Department of Plant Science and Landscape Architecture, 1390 Storrs Road, U-4163, University of Connecticut, Storrs, Connecticut, 06269, United States of America; 2 USDA, Agricultural Research Service, Vegetable Crops Research Unit, Department of Horticulture, University of Wisconsin, 1575 Linden Drive, Madison, Wisconsin, 53706, United States of America; Beijing Forestry University, CHINA

## Abstract

Switchgrass (*Panicum virgatum* L.) is a North American grass that exhibits vast genetic diversity across its geographic range. In the Northeastern US, local switchgrass populations were restricted to a narrow coastal zone before European settlement, but current populations inhabit inland road verges raising questions about their origin and genetics. These questions are important because switchgrass lines with novel traits are being cultivated as a biofuel feedstock, and gene flow could impact the genetic integrity and distribution of local populations. This study was designed to determine if: 1) switchgrass plants collected in the Long Island Sound Coastal Lowland coastal Level IV ecoregion represented local populations, and 2) switchgrass plants collected from road verges in the adjacent inland regions were most closely related to local coastal populations or switchgrass from other geographic regions. The study used 18 microsatellite markers to infer the genetic relationships between 122 collected switchgrass plants and a reference dataset consisting of 28 cultivars representing ecotypes, ploidy levels, and lineages from North America. Results showed that 84% of 88 plants collected in the coastal plants were most closely aligned with the Lowland tetraploid genetic pool. Among this group, 61 coastal plants were similar to, but distinct from, all Lowland tetraploid cultivars in the reference dataset leading to the designation of a genetic sub-population called the Southern New England Lowland Tetraploids. In contrast, 67% of 34 plants collected in road verges in the inland ecoregions were most similar to two Upland octoploid cultivars; only 24% of roadside plants were Lowland tetraploid. These results suggest that cryptic, non-local genotypes exist in road verges and that gene flow from biofuels plantations could contribute to further changes in switchgrass population genetics in the Northeast.

## Introduction

Switchgrass (*Panicum virgatum* L.) is a native, perennial grass across Eastern and Central North America [1,2]. Research has generally divided switchgrass into two ecotypes; individuals in the Lowland ecotype are usually tetraploid, while those in the Upland ecotype are either tetraploid or octaploid [3,4]. Upland plants are typically associated with dry habitats and colder northern latitudes, while Lowland plants are found in moist habitats in warmer southern latitudes [4,5]. However, a more complex picture of ecotypes is emerging including recognition of an Eastern coastal ecotype growing in the salt spray zone, dunes and salt marshes along the Gulf Coast and Atlantic Ocean [5,6]. Recent botanical surveys in the Northeastern portion of the distribution range found numerous switchgrass populations in road verges as much as 188 km from the coast raising questions about their origins, genetics and distribution in current and future climate regimes [7,8].

Switchgrass ‘source identified’ cultivars have been grown for many purposes including livestock forage, wildlife habitat, prairie restoration, ornamental gardens, roadside plantings and erosion control. Recent efforts to develop renewable energy have led to the genetic modification of switchgrass as a dedicated biofuels feedstock. However, the adaptability of switchgrass and the introduction of novel traits have generated concern about new weed problems or invasive species [9,10]. Conventional breeding and genetic engineering (GE) have been used to create a model biofuels feedstock [11,12] and approve a GE switchgrass for cultivation in the U.S. [13]. These advances have created a need for scientific information to develop predictive ecological risk assessments and effectively confine experimental field trials. An ecological risk is defined as the product of a hazard (a specific adverse impact to the environment) and an exposure (a mechanism/route by which the hazard is experienced) [14–16]. Ecological risk assessments for GE crops utilize information about the crop species (e.g. reproductive biology), novel genes and traits, and the receiving environment (e.g. native plants communities) to support regulatory decision-making and risk management activities. The potential impacts of switchgrass to valued environmental endpoints (e.g. communities, ecosystems) could include: 1) the development of weedy or invasive switchgrass populations that require management; 2) the reduction or extinction of local switchgrass populations due to gene flow or direct competition with GE switchgrass; 3) loss of genetic diversity or genetic resources in local populations; 4) changes in switchgrass distribution; 5) interspecific gene flow to native or non-native *Panicum* species with ecological impacts; 6) undesirable changes in natural or managed plant communities; 7) negative effects on non-target organisms [9,10,17–21]. Indeed, switchgrass has a number of traits that increase the likelihood of risk including a perennial lifecycle, a high degree of adaptability (e.g. cold hardiness, drought tolerance) and minimal domestication [3,22–24]. Some related *Panicum* taxa are already weeds (e.g. *P*. *capillare* and *P*. *dichotomiflorum*). The potential for gene flow is increased by large panicles with a long period (4–5 weeks) of asynchronous pollen release; obligate out-crossing, small seeds that are easily dispersed and shared habitat with sexually-compatible wild relatives [3,22–24]. A modeling study predicted that viable, wind-blown switchgrass pollen could move up to 3.5–6 km under normal summer wind conditions in the Northeastern U.S. [22]. Collectively, these factors highlight the importance of risk assessment research and careful management.

Road verges are interconnected, linear habitats that can alter the distribution of native and introduced plant species [25,26]. They are among the most common habitat types in human-impacted ecosystems with differences from surrounding communities involving soil drainage, compaction (higher bulk density) and pH, as well as light availability and disturbance events (e.g. mowing) [26,27]. While the idea that road corridors can aid dispersal is widely accepted, there is no consensus about their effects on individual plant species. A study on invasive *Phragmites australis* concluded that the development of a highway network contributed to its inland expansion [28], whereas a study on weedy *Raphanus raphanistrum* reported that road verges did not act as dispersal corridors [29]. Relatively few studies have examined the function of road verges with regard to changes in native plant distribution [25]. A study on *Phragmites australis* chloroplast DNA concluded that the introduction of a non-native haplotype to North America had diminished the native populations while increasing non-native distribution [30].

This study was designed to support ecological risk assessments for switchgrass biofuels plantations by determining if: 1) switchgrass plants collected in the Long Island Sound Coastal Lowland (LISCL) Level IV ecoregion represented local populations, and 2) switchgrass plants collected from road verges in the adjacent inland ecoregion were most closely related to local populations or genotypes introduced from other geographic regions. This constitutes the first study on switchgrass population genetics in this part of its distribution range.

## Materials and Methods

**Fig 1:**
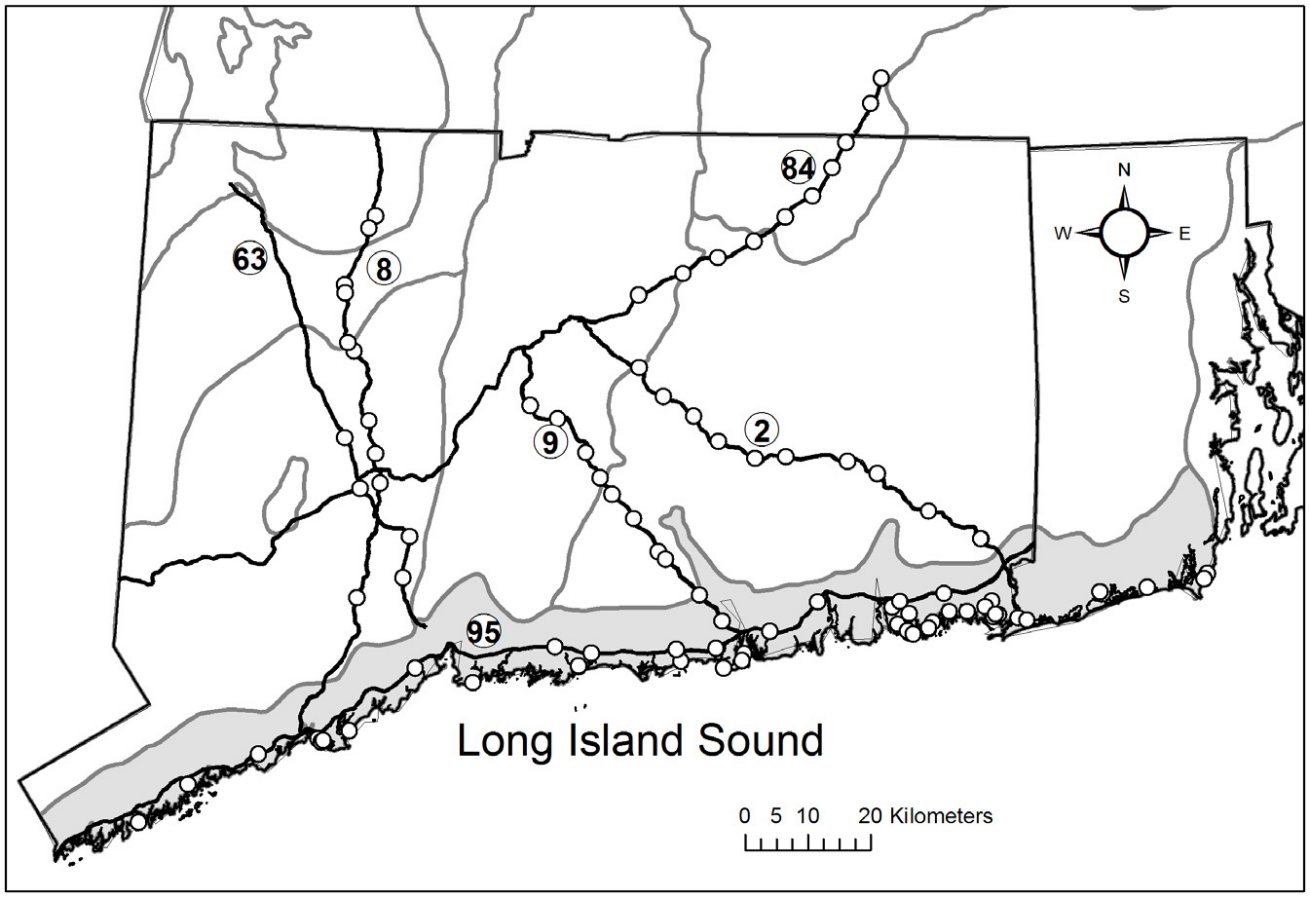
Map of the study site. Switchgrass collection sites (open circles). Black lines represent borders of Connecticut and Rhode Island and six roads identified as Routes 63, 8, 9, 95, 84,and 2. Grey lines represent the borders of Level IV sub-ecoregions. The shaded area represents Level IV sub-ecoregion 59g Long Island Sound Coastal Lowland.

Switchgrass samples were collected from public road verge right-of-ways or state parks with permission from the Connecticut State Department of Energy and Environmental Protection. The field work did not affect endangered or protected species. The study site (Fig 1) was located within the western boundary of (-73.500621), eastern boundary of (-71.468331), northern boundary of (42.111813), and southern boundary of (41.04363). Spatial information about collection sites was obtained using a Juno SB GPS unit (Trimble Navigation Limited, Westminster, CO, USA) and processed using ArcGIS 10.2 (ESRI, Redlands, CA). Spatial layers for ecoregions and roads were obtained from the United States Environmental Protection Agency (EPA) and the Connecticut Department of Energy and Environmental Protection respectively [31,32].

The collection of switchgrass samples was informed by two previous studies on its habitats and distribution in two Level III Ecoregions: the Northeast Coastal Zone, and Northeastern Highland Zone [7,8]. For the purposes of this study, two distinct regions were identified for switchgrass collection. The ‘coastal’ samples were collected from the Level IV ecoregion 59g Long Island Sound Coastal Lowland (LISCL). The ‘inland’ road verge samples were collected from Level IV ecoregions including: 59a Connecticut Valley, 59b Lower Worcester Plateau/Eastern Connecticut Upland, 59c Southern New England Coastal Plains and Hills, 58d Lower Berkshire Hills, and 58e Berkshire Transition. In the LISCL coastal ecoregion, plants (n = 88) were collected at 37 locations in three habitat types previously shown to support switchgrass: semi-natural, human impacted, and road verges including Interstate 95 [7]. At sites with larger coastal populations, three individuals were collected at least 2 m apart to avoid sampling more than once from a single genet. All three individuals were only used in the first STRUCTRE analysis; subsequent analyses of the Simple Sequence Repeat (SSR) dataset included only one individual from the 37 coastal sites. Preliminary switchgrass surveys were conducted on 14 roads in a north-south orientation perpendicular to the Long Island Sound coastline; five roads (Routes 63, 8, 9, 84, 2) were selected for sampling with 34 individuals collected at least 1.6 km apart and within 3m of the pavement.

Eighteen SSR markers were used to amplify the DNA from 122 collected switchgrass plants and 61 individuals representing 25 cultivars (Table 1). These results were aligned and combined with re-analysis of electrophaerograms from samples [24] consisting of 182 individuals from 18 cultivars (http://hdl.handle.net/11134/20003:66). By combining the two datasets, a large reference dataset was created that included 28 cultivars representing switchgrass ecotypes, ploidy levels, and lineages from North America (Table 1). None of the cultivars originated from the study site. Cultivar seed was acquired from the USDA National Genetic Resources Program (www.ars-grin.gov), Ernst Seeds (Meadville, PA, USA), or Sharpe Brothers Seed Company (Clinton, MO, USA). While any cultivar could be used as an ornamental plant, five ornamental cultivars were chosen for this study because they were recently developed for ornamental traits (e.g. red leaves), had not been used in any previous genetic studies, and are popular garden plants. Individuals were obtained as vegetative propagules from Broken Arrow Nursery (Hamden, CT, USA). All plants were grown in the University of Connecticut greenhouses. The geographic origin of cultivars and lineage designations are as described in a previous review of switchgrass genetics [33]. Lineages represented in this study include: upland tetraploids A and C, upland octoploid (U8x) A and B, and lowland tetraploid (L4x) A, C, and D.

**Table 1:** Switchgrass accessions in the reference dataset.

Cultivar	Origin	Ecotype	Ploidy	Lineage	TE	RE	Source
Dakotah	North Dakota	Upland	4x	U4xA	1	16	GRIN
Summer	Nebraska	Upland	4x	U4xC	1	16	Ernst
Cave-in-rock	Illinois	Upland	8x	U8xA	4	16	GRIN
Shawnee	Illinois	Upland	8x	U8xA	2	4	GRIN
Shelter	West Virginia	Upland	8x	U8xA	18	5	Ernst
Carthage	North Carolina	Upland	8x	U8xB	1	8	Ernst
Pathfinder	Nebraska	Upland	8x	U8xB	1	8	GRIN
Forestburg	South Dakota	Upland	8x	U8xB	1	17	GRIN
Sunburst	South Dakota	Upland	8x	U8xB	-	9	GRIN
Trailblazer	Nebraska	Upland	8x	U8xB	1	8	GRIN
Blackwell	Oklahoma	Upland	8x	U8xB	-	16	Sharp
Caddo	Oklahoma	Upland	8x	-	1	-	Ernst
High Tide	Maryland	Upland	-	-	1	-	Ernst
Southlow	Michigan	Upland	-	-	1	-	Ernst
Miami	Florida	Lowland	4x	L4xA	1	2	GRIN
Wabasso	Florida	Lowland	4x	L4xC	1	6	GRIN
Stuart	Florida	Lowland	4x	L4xC	1	2	GRIN
Alamo	Texas	Lowland	4x	L4xD	4	16	Sharp
Kanlow	Oklahoma	Lowland	4x	L4xD	1	16	GRIN
SG5	-	Lowland	4x	L4xD	-	8	GRIN
Timber	New Jersey	Lowland	4x	L4xD	11	9	GRIN
BoMaster	North Carolina	Lowland	4x	-	2	-	Ernst
Performer	North Carolina	Lowland	4x	-	2	-	Ernst
Ruby Ribbions*	-	-	-	-	1	-	BAN
Northwind*	-	-	-	-	1	-	BAN
Shenandoah*	-	-	-	-	1	-	BAN
Dallas Blue*	-	-	-	-	1	-	BAN
Haron Salstius*	-	-	-	-	1	-	BAN
Inland Roads	Conneticut / Massachusetts	-	-	-	34	-	-
Coastal	Conneticut / Rhode Island	-	4x	-	88	-	-

Information about switchgrass cultivar geographic origin, ecotype, ploidy and lineage were as previously published [32] and as described in the Methods. Numbers in the Tissue Extracted (TE) column represent the number of plants with DNA extraction and SSR analysis. Numbers in the Reanalyzed Electropherograms (RE) column represent the number of electropherograms re-analyzed and integrated into this study. The symbol (-) means that information was not available and (*) indicates ornamental cultivars. Abbreviations for propagule sources: USDA National Genetic Resources Program (GRIN); Ernst Seeds (Ernst); Sharpe Brothers Seed Company (Sharpe); and Broken Arrow Nursery (BAN).

DNA extraction involved processing 100 mg frozen leaf tissue with a TissueLyser II (Qiagen, Valencia, CA, USA) followed by DNeasy Plant Mini kits (Qiagen, Valencia, CA, USA). Eighteen previously-published primer pairs [24] were synthesized by the University ofWisconsin BiotechnologyCenter and used in PCR according to published protocols [24]. The SSR primer pairs used were SWW 112, 151, 185, 432, 438, 439, 593, 651, 664, 686, 2309, 2312, 2341, 2385, 2394, 2415, 2416, 2431 [24].

DNA fragment analysis was performed by Cornell University (Institute of Biotechnology, Ithaca, NY) using an ABI 3730xl DNA analyzer (Applied Biosystems, Foster City, CA, USA). Genotypes were derived from scoring of alleles using GeneMarker v 1.95 (Softgenetics, State College, PA, USA). Because switchgrass is polyploid, SSR data was transformed from binary data to fragment sizes and analyzed in relation to the corresponding primer pair using polysat version 1.3–2 in R [34]. Principle coordinate analysis (PCA) was conducted in polysat using Bruvo distances [35], polymorphic information content (PIC) was calculated usingPICcalc [36], Analysis of Molecular Variance (AMOVA) was performed in GenAlEx [37], and Bayesian inference of genotypic groups was conducted through STRUCTURE 2.3.4 [38] using the ‘admixture model’ and 25,000 Markov chain Monte Carlo iterations with 10,000 burn-in iterations and 10 replicates per run. Using the admixture model, q was defined as the proportion of an individual’s ancestry in one of K populations. Optimal K values for STRUCTURE output were calculated using Evanno's delta K implemented in Structure Harvester [39]. Clumpak and Distruct were used to produce publication quality figures from STRUCTURE output [40,41].

## Results

### Switchgrass Genetic Pools

**Fig 2:**
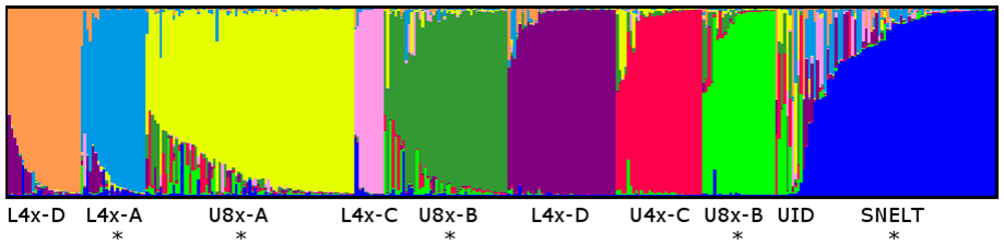
STRUCTURE analysis of SSR markers from all switchgrass cultivars and collected plants (n = 365). Individuals are shown as a vertical line representing the membership fractions for each of the K populations and sorted according to q-value (K = 9). Switchgrass lineages abbreviated as shown in [42]. Other abbreviation: Southern New England Lowland Tetraploid, SNELT; UID, Unidentified. Genotype groups with an asterisk (*) include one or more individuals collected from the LISCL coastal ecoregion or inland road verges.

In order to infer switchgrass population structure and the membership of each collected sample, a large reference dataset was created by analyzing SSR markers in plants representing a wide array of ecotypes, ploidy levels, and lineages. STRUCTURE analysis (k = 9) of the reference dataset (n = 365) confirmed the population membership assignments given to cultivars in previous studies (Fig 2) [24,42,43]. Switchgrass plants collected from the inland road verges and LISCL sites grouped into four genetic clusters (Fig 2): 29 individuals were associated with the U8x-A lineage as represented by ‘Cave-in-Rock’ and ‘Shelter’; 19 individuals were linked with the L4x-A lineage as represented by ‘Miami’; one individual was U8x-B as represented by ‘Pathfinder’, ‘Shawnee’, ‘Blackwell’, ‘Carthage’, and ‘Trailblazer’; 63 collected plants were distinct from all cultivar comparators. This group was named the Southern New England Lowland Tetraploid (SNELT). Ten collected plants could not be assigned to a group.

### Performance of SSR Markers

Although none of the SSR markers yielded unique alleles for the SNELT group, switchgrass plants collected from the inland road verges and LISCL sites generated 156 distinct alleles (mean 30.4, range = 8–48) with a mean 8.7 alleles per primer pair (range = 4–23) based on the 18 SSR primer pairs (Table 2). The largest number of private alleles was found in the U8x and L4x cultivar groups; switchgrass collected across the whole study site had 3–4 private alleles, and two U4x cultivars had no private alleles. The PIC value across all primer pairs (loci) ranged from 0.79–0.96 with an mean of 0.91; this was higher than previously reported for a larger set of 55 primer pairs (mean = 0.66, [24]) or 19 primer pairs (mean = 0.71,[42]). Thus, the 18 SSR loci used in this switchgrass study were slightly more informative than previous studies.

**Table 2:** Performance of 18 SSR markers in switchgrass.

Study Samples	All individuals	U4x	U8x	L4x	Coastal	Inland
**Number of Alleles**	156	87	129	115	104	112
**Number of Alleles Freq. ≥ 5%**	97	73	95	78	66	90
**Number of Private Alleles**	-	0	8	8	4	3

Performance of 18 SSR markers in switchgrass DNA from U4x, U8x and L4x cultivars and collected plants (coastal ecoregion and inland ecoregion).

Analysis of molecular variance (AMOVA) was conducted within and among plants collected in the coastal and inland ecoregions, and within and among the 18 cultivars in the reference dataset. Results indicated that the majority of genetic diversity was detected within rather than between (among) ecoregions, cultivars, or ecotype/ploidy groups (Table 3).

**Table 3:** Analysis of molecular variance.

Source of Variation	df	SS	MS	%	p
(1) Collected Individuals					
Among Coastal or Inland	1	204.88	204.88	0.233147	<0.001
Within Coastal & Inland	123	1496.555	12.16711	0.766853	<0.001
Total	124	1701.435		1	
(2) Cultivars					
Among Ecotype	2	741.7149	370.8575	0.231414	<0.001
Among Cultivars	15	769.6335	51.3089	0.171198	<0.001
Within Cultivars	213	2434.237	11.42834	0.597388	<0.001
Total	230	3945.585		1	

Analysis of molecular variance (AMOVA): 1) within and among plants collected in the coastal or inland ecoregion, and 2) within and among all cultivars in the reference dataset.

Switchgrass lines have been selected for ornamental traits such as red or blue-green leaf color and five such cultivars were analyzed with SSR markers because these garden plants could potentially escape cultivation (Table 1). SSR marker analysis showed that ‘Dallas Blue’ and ‘Northwind’ had primary membership probability with the L4x-A cultivar ‘Miami’. ‘Haron Salstius’ grouped with the U8x-B cultivars ‘Sunburst’ and ‘Forestburg’. Cultivars ‘Ruby Ribbons’ and ‘Shenandoah’ showed mixed genetic assignment. However, there was no evidence that these cultivars had contributed to roadside or coastal populations in this study.

### Long Island Sound Coastal Lowland Ecoregion (LISCL)

**Fig 3:**
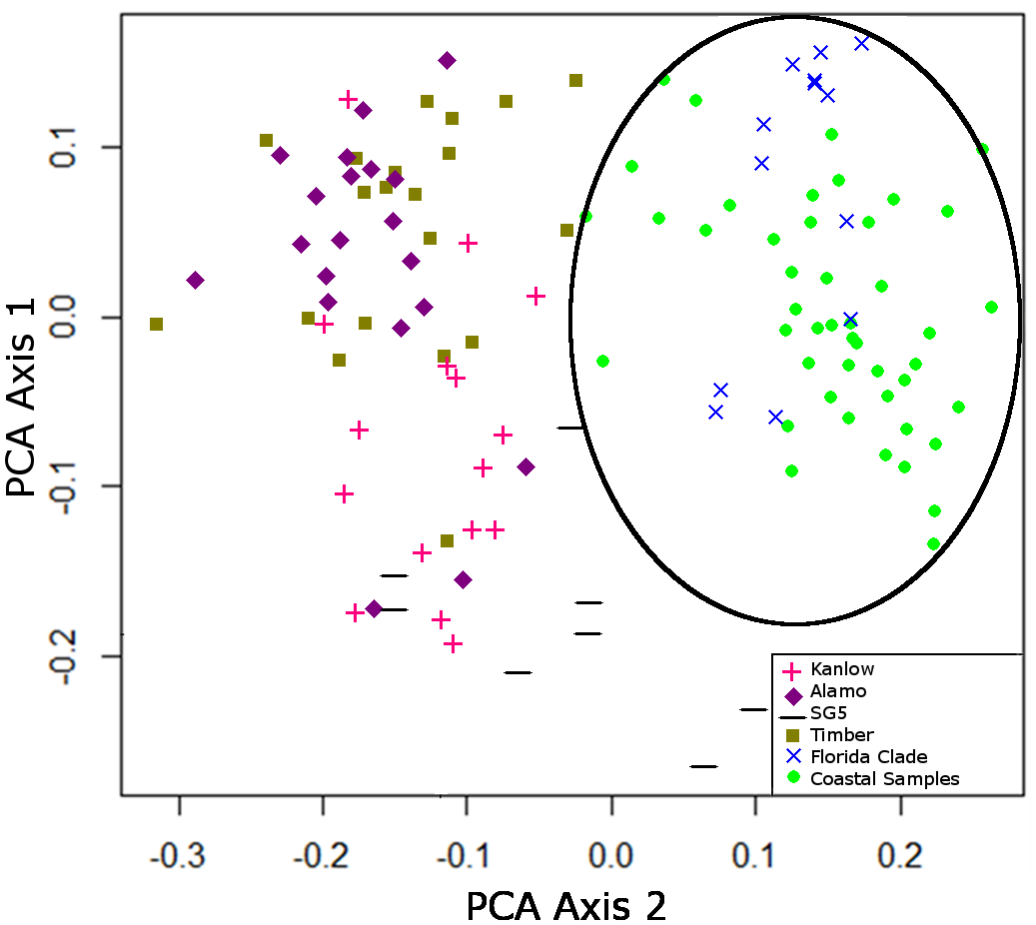
Principle Coordinate Analysis (PCA) of SSR markers from L4x cultivars and plants collected in the LISCL coastal ecoregion (n = 124). The circle indicates the clustering of collected LISCL plants with three L4x cultivars (‘Miami’, ‘Wabasso’, ‘Stuart’) representing the Florida Clade. Colors indicate: ‘Kanlow’ (red+), ‘Alamo’ (purple♦), ‘SG5’ (black-), ‘Timber’ (olive green■), Florida Clade cultivars (blue×), and coastal samples (light green●).

Of the 88 switchgrass plants collected in the LISCL ecoregion, 84% (74 plants) were assigned to the L4x genotype as the SNELT group or the L4x-A lineage (Fig 2). U8x plants comprised 8% (7 plants) of the LISCL individuals, and 8% plants (7 plants) were unidentified. Flow cytometry was conducted on 20 SNELT individuals and all were classified as tetraploid when compared to control L4x and U8x cultivars [44] providing further support for the conclusion that SNELT plants were L4x. PCA analysis was conducted on a subset of the data to compare the LISCL L4x individuals to seven L4x cultivars including three cultivars (‘Miami’, ‘Wabasso’, ‘Stuart’) that represent two lineages in the ‘Florida Clade’ [24,42] (Fig 3). The collected L4x plants grouped together and overlapped with the Florida Clade cultivars, but were distinct from L4x cultivars originating in Texas, Oklahoma, or New Jersey.

**Fig 4:**
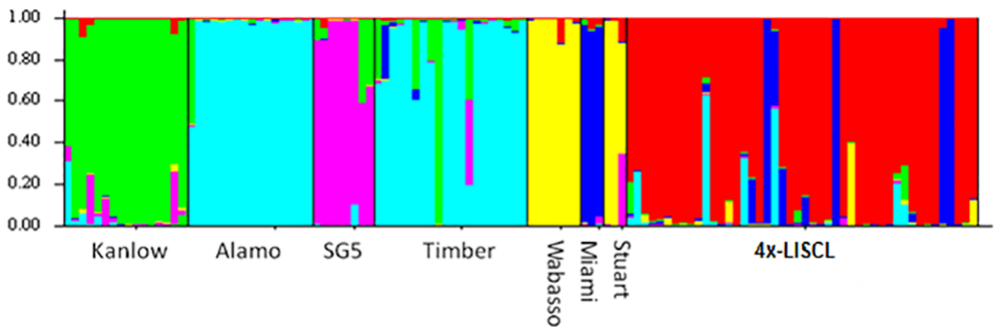
STRUCTURE analysis of SSR markers from L4x LISCL plants (n = 46) and seven L4x cultivars. Individuals are shown as a vertical line representing the membership fractions for each of the K populations and sorted according to q-value (K = 6).

STRUCTURE analysis of the coastal L4x plants using a single individual from each collection site (n = 46) and seven L4x cultivars (Fig 4) showed a slightly different picture. Forty plants were SNELT, four plants were grouped with ‘Miami’, one individual had affiliation with ‘Alamo’ or ‘Timber’, and one individual could not be assigned to a particular group. In general, SNELT plants were collected from Atlantic coastal habitats including dunes, the edges of salt water marshes and riparian habitats previously reported as typical for the Lowland ecotype [4,5,45]. This provided further evidence that a local L4x genotype was identified in the study site.

### Switchgrass from Inland Ecoregion Road Verges

**Fig 5:**
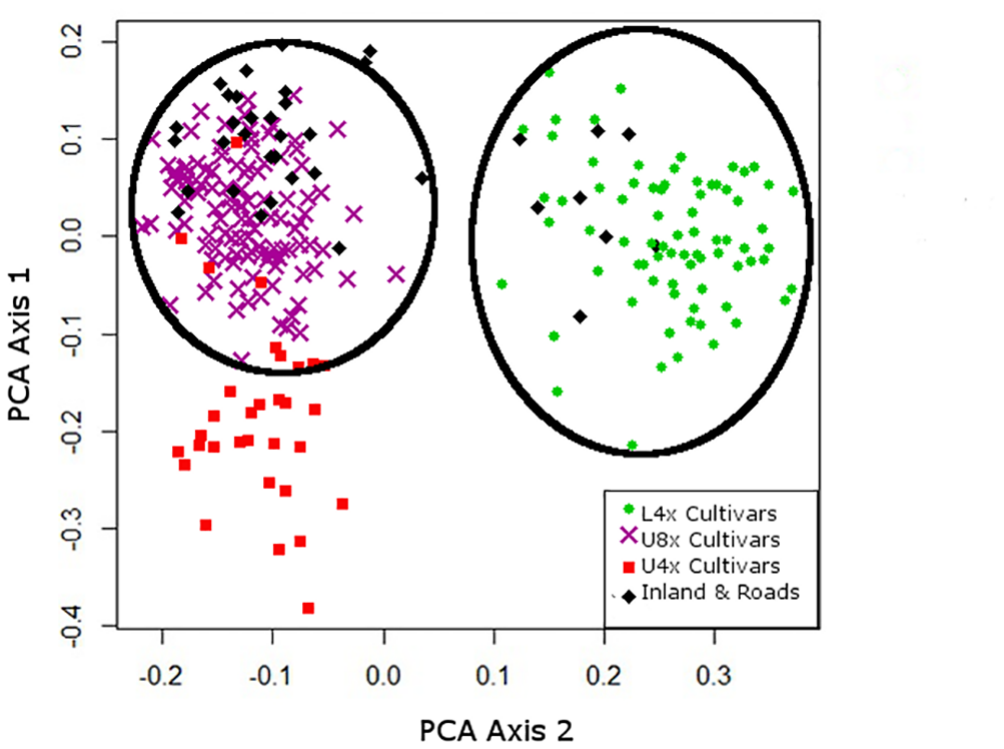
Principle Coordinate Analysis (PCA) of SSR markers from switchgrass collected from inland road verges and 18 U4x, U8x, and L4x switchgrass cultivars (n = 265). Circles indicate inland road plants clustering with either U8x or L4x but not U4x cultivars. Colors indicate: U4X cultivars (Red■), U8X cultivars (PurpleX), L4X cultivars (Green●), plants from inland road verges (Black♦).

**Fig 6:**
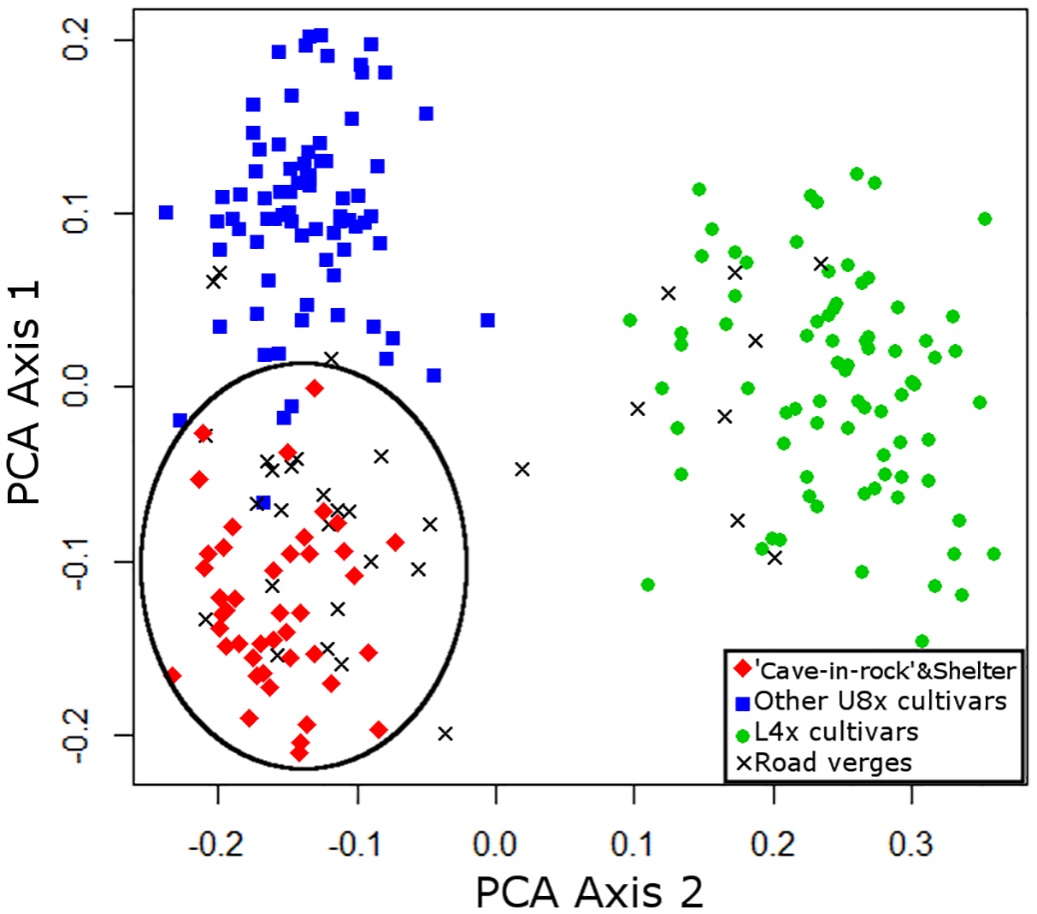
Principle Coordinate Analysis (PCA) of SSR markers from switchgrass collected from inland road verges and 16 cultivars representing U8x and L4x genotypes (n = 231). The circle indicates the grouping of road verge samples with cultivars ‘Cave-in-Rock’ and ‘Shelter’. Colors indicate: ‘Cave-in-rock’ and ‘Shelter’ (Red♦), Other U8X cultivars (Blue■), L4X cultivars (Green●), plants from inland road verges (BlackX).

Analysis of switchgrass plants collected from inland ecoregion road verges (n = 34) using PCA showed that 76% (26 plants) grouped with Upland cultivars (U8x and U4x), while 22% (8 plants) grouped with L4x cultivars (Fig 5). None of the inland road verge plants were associated with U4x cultivars, so these cultivars were dropped from further analysis. PCA analysis was conducted with inland roadside plants, U8x cultivars, and L4x cultivars (Fig 6). Twenty-two plants grouped with two U8x-A cultivars: ‘Cave-in-rock’ and ‘Shelter’. As in other studies, the SSR markers were unable to distinguish between these two cultivars from the Ohio River Valley and Central Appalachian Mountain region, possibly because ‘Shelter’ was derived from ‘Cave-in-Rock’ [24,46,47]. Three roadside plants grouped with all other U8x cultivars, eight individuals grouped with L4x cultivars, and two did not have a clear genetic assignment.

### Spatial Distribution of Switchgrass Genotypes

**Fig 7:**
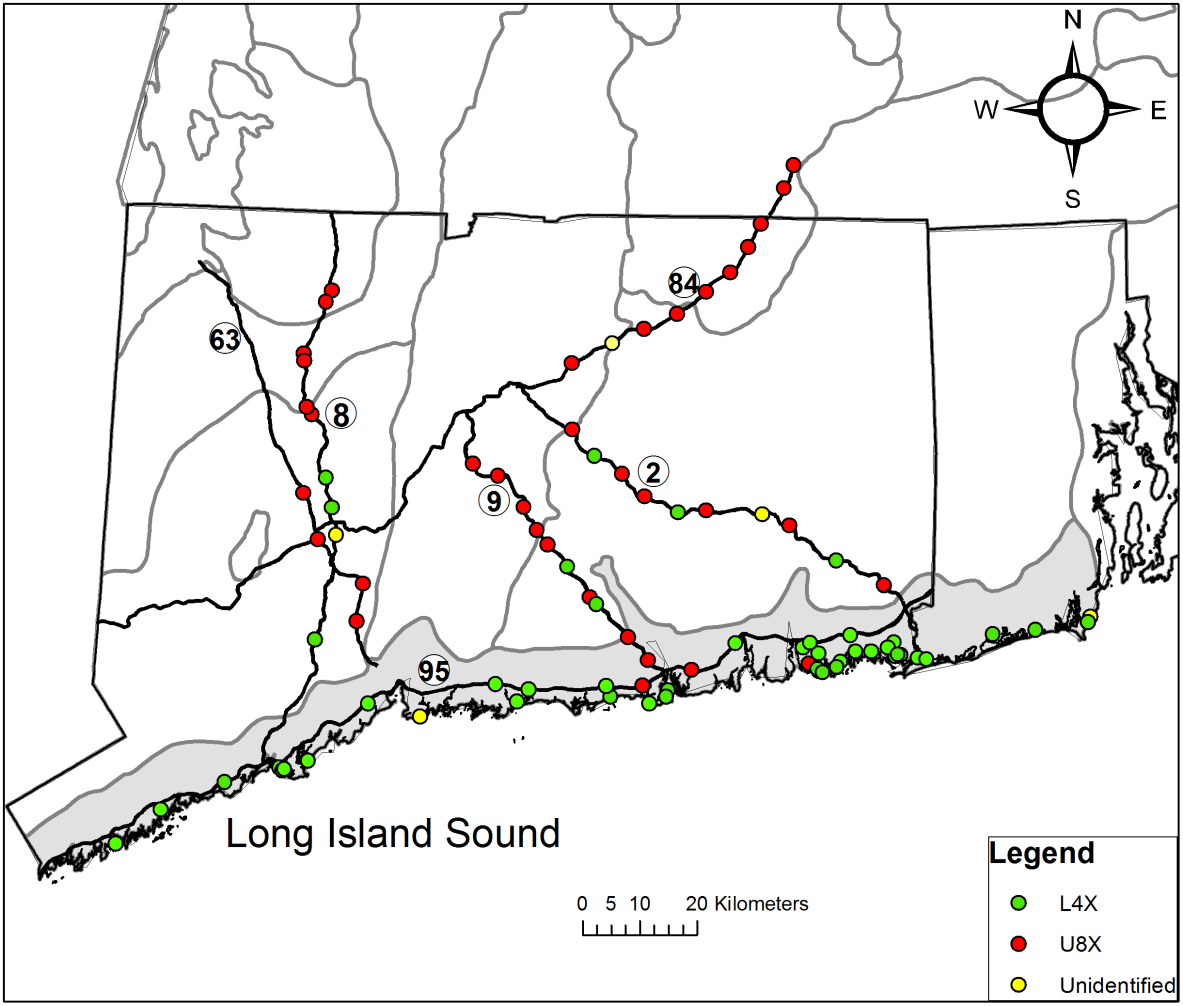
Map of study site showing distribution of switchgrass genotypes. Black lines represent borders of Connecticut and Rhode Island and six roads identified as Routes 63, 8, 9, 95, 84, and 2. Grey lines represent the borders of Level IV sub-ecoregions. The shaded area represents sub-ecoregion 59g Long Island Sound Coastal Lowland (LISCL). Green dots represent plants identified as SNELT or other L4x genotypes; red dots represent plants with an U8x genotype; yellow dots represent individuals that could not be assigned to a specific group.

A general north-south trend was observed when the L4x and U8x switchgrass genotypes were mapped to their collection sites (Fig 7, Table 4). The LISCL southern coastal region had a higher percentage of L4x plants including the local SNELTs, while the northern inland region had a higher percentage of individuals grouped with U8x cultivars. However, the ecoregions were not homogeneous with regard to genetic pool membership. For example, two U8x individuals were found close to the Long Island Sound (7 km and 13 km from the Long Island Sound), and two SNELT individuals were found far inland (40 and 44 km from the Long Island Sound). A few plants without clear genetic assignment were found in both regions; three in inland road verges and seven in the coastal zone. Further research would be required to determine if these individuals were hybrids.

**Table 4:** Percentage of switchgrass ecotypes in coastal or inland regions.

	L4X	U8X	Unidentified
Long Island Sound Coastal Lowland	84%	8%	8%
Inland road verges	24%	67%	9%

Percentage of switchgrass plants collected in the Long Island Sound Coastal Lowland region or the inland region road verges identified as L4X or U8X based on STRUCTURE analysis (Fig 2). Individuals without a clear genetic assignment were put in the Unidentified group.

## Discussion

### Identification of a Local Switchgrass Genotype

This was the first study of switchgrass genetics in the Long Island Sound Coastal Lowland (LISCL) ecoregion of Connecticut and Rhode Island where its natural distribution has been described as a narrow zone along the Atlantic coast [6]. Although this coastal habitat has been severely altered by human activity, SSR markers identified a local L4x genotype designated as the Southern New England Lowland Tetraploid (SNELT). The SSR markers showed that SNELT individuals were distinct from known genetic pools and L4x cultivars sold by seed companies including the older L4x cultivars ‘Alamo’ (released 1978) and ‘Kanlow’ (1963), and newer L4x cultivars ‘Timber’ (2009), ‘BoMaster’ (2006), and ‘Performer’ (2006) [33]. In PCA and STRUCTURE analysis, SNELT plants also remained distinct from three L4x cultivars associated with the Florida Clade (‘Miami’, ‘Wabasso’, and ‘Stuart’ released around 1996) [42]. The presence of individuals that grouped with the L4x ‘Miami’ cultivar could not be attributed to recent human introduction since this germplasm is not common in commercial seed mixes. The identification of a unique and localized L4x genotype is similar to results from previous studies that examined Lowland switchgrass lineages [42,48]. For example, Lu et al. [48] collected switchgrass from New York including Long Island and suggested that these individuals belonged to a “Lowland 4x Northeast” group. However, this study could not make a connection with the L4x Florida Clade identified by Zhang et al [42] because it did not include accessions from the Southeastern Atlantic or Gulf Coast states (Florida, South Carolina, North Carolina). The study by Zhang et al. [42] included samples from the Southeastern region, but the northern edge of their collections was New York City and New Jersey. Thus, our results support previous evidence that the Northeastern coastal lineage is L4x, and that these plants are probably derived from refugia in the Southeastern Atlantic or Gulf Coast region during the last ice age [5,42,48,49]. In contrast, Cortese et al. [50] collected one switchgrass population in Brooklyn, New York and reported that their plants were most similar to Upland cultivars. Regional genetic studies have also reported localized switchgrass genotypes in the Southeast and Midwestern US [47,49,51,52]. Our study supports an increasingly complex picture of switchgrass genetics by identifying a distinct Northeastern coastal genotype associated with the salt spray zone, dunes, salt marshes and riparian habitats in the Atlantic coastal ecoregion.

### Spatial Distribution of Genotypes

A map of switchgrass genotypes showed a north-south gradient with L4x plants dominant near the coast and U8x plants more abundant towards the northern edge of the study site (Fig 7, Table 4). STRUCTURE analysis indicated that 67% of the inland switchgrass collected from road verges were most similar to U8x-A cultivars ‘Cave-in-Rock’ and ‘Shelter’ which originated from the Ohio River Valley or the Central Appalachian Mountain Region [42]. Only 24% of plants in the inland road verges were assigned the L4x genotype. Thus, current roadside populations could not be explained by dispersal of seed or vegetative propagules from coastal populations along road corridors. The simplest explanation for the observed distribution pattern is that ‘Cave-in-Rock’ or ‘Shelter’ were introduced through human activity. However, to the best of our knowledge, the state transportation department has recommended only low-growing, non-native turfgrass species and has never promoted switchgrass in roadside plantings. Conversely, a recent regional publication recommended switchgrass for roadsides due to its ecological functions and adaptability [53]. U8x cultivars could also have been introduced for gardens, wildlife habitat, erosion control, livestock forage, or other purposes followed by seed dispersal and establishment in suitable road verge habitat. While some form of human activity probably explains the presence of U8x cultivars, these cultivars might have benefited from their increased cold hardiness and decreased mortality in severe winter temperatures [54,55]. A recent modeling study showed that minimum winter temperature was an important explanatory variable in switchgrass distribution in the Northeastern US [8]. Temperature in late summer plays a role in switchgrass flowering and reproduction, so it is possible that inland temperatures were more favorable for U8x plants than other genotypes [56]. U8x plants could also have been favored by well-drained soils in road verges [45]. An alternative explanation for the north-south distribution pattern was that this study site encompassed a natural transition zone between L4x and U8x genotypes in North America. A theoretical map of ecotype distribution [33] included a broad Upland-Lowland transition zone that encompassed the study site, but this is an unlikely explanation because: 1) the dominant inland habitat types and plant communities (e.g. forest) do not include switchgrass [7,8]; 2) the botanical literature identifies the pre-settlement distribution as a narrow zone adjacent to the coast, 3) most road verges did not exist until recently, and 4) the U8x plants in this study were either identical to or very closely related to the relatively common cultivars ‘Cave in Rock’ and ‘Shelter’ originating from Illinois.

### Implications for Conservation and Ecological Risk Assessment

The identification of the SNELT genotype indicates the importance of documenting local switchgrass populations and preserving genetic resources through seed collections and other actions. To support future research on switchgrass genetic resources, one SNELT plant was given the name ‘Hammonasset’ and contributed to a DNA sequencing project (Joint Genome Institute, Project #1030572, http://genome.jgi-psf.org/Panvirsequencing_24/Panvirsequencing_24.info). The abundance of U8x plants in inland road verges suggested that cryptic populations of non-local genotypes were well established in the study site. Additional research should examine the probability of these plants increasing their distribution range and abundance under current or future climate regimes. With regards to future crop-to-wild gene flow, both L4x and U8x switchgrass populations exist and could be receiving populations for pollen from GE biofuels fields. If preservation of genetic resources is an objective, the use of U8x genotypes in switchgrass biofuels plantations would decrease the likelihood of pollen-mediated gene flow to local L4x SNELT populations in this region.
